# Effects of transcranial alternating current stimulation on motor performance and motor learning for healthy individuals: A systematic review and meta-analysis

**DOI:** 10.3389/fphys.2022.1064584

**Published:** 2022-11-18

**Authors:** Kun Hu, Ruihan Wan, Ying Liu, Maolin Niu, Jianrui Guo, Feng Guo

**Affiliations:** ^1^ College of Human Kinesiology, Shenyang Sport University, Shenyang, Liaoning, China; ^2^ College of Rehabilitation Medicine, Fujian University of Traditional Chinese Medicine, Fuzhou, Fujian, China; ^3^ Department of Rehabilitation Medicine, Nanfang Hospital, Southern Medical University, Guangzhou, Guangdong, China

**Keywords:** transcranial alternating current stimulation, motor performance, motor learning, healthy individuals, frequency specificity

## Abstract

**Objective:**Previous behavioral studies have reported the potential of transcranial alternating current stimulation in analyzing the causal relationship between neural activity and behavior. However, the efficacy of tACS on motor performance and learning in healthy individuals remains unclear. This systematic reviewexamines the effectiveness of tACS on motor performance and motor learning in healthy individuals.

**Methods:** Literature was systematically searched through the Cochrane Library, PubMed, EMBASE, and Web of Science until 16 October 2022. Studies were eligible for review if they were randomized, parallel, or crossover experimental designs and reported the efficacy of tACS on motor performance and motor learning in healthy adults. Review Manager 5.3 was used to evaluate the methodological quality and analyze the combined effect.

**Results:** Ten studies (270 participants) met all the inclusion criteria. The results showed that motor performance was not significantly greater than that with sham tACS stimulation [I^2^ = 44%, 95% CI (–0.01, 0.35), *p* = 0.06, standardized mean difference = 0.17], whereas motor learning ability improved significantly [I^2^ = 33%, 95% CI (−1.03, −0.31), *p* = 0.0002, SMD = −0.67]. Subgroup analysis found that gamma bend tACS could affect the changes in motor performance (I^2^ = 6%, 95% CI (0.05, 0.51), *p* = 0.02, SMD = 0.28), and online tACS did as well [I^2^ = 54%, 95% CI (0.12, 0.56), *p* = 0.002, SMD = 0.34].

**Conclusion:** The results showed that tACS effectively improves motor performance (gamma band and online mode) and motor learning in healthy individuals, which indicates that tACS may be a potential therapeutic tool to improve motor behavioral outcomes. However, further evidence is needed to support these promising results.

**Systematic Review Registration:** PROSPERO, identifier CRD42022342884

## 1 Introduction

Motor performance and motor learning play a leading role in improving the quality of daily life in healthy adults, including physiological aspects, psychological health, and cognitive performance (Li et al., 2022; Orssatto et al., 2022; Wiegel et al., 2022; Zhou et al., 2022). Studies in the field of sports sciences have indicated that transcranial alternating current stimulation (tACS) can be a promising noninvasive brain stimulation tool to improve motor performance and motor learning, slowing declines in physical function (Urrútia and Bonfill, 2010; Antal and Herrmann, 2016).

TACS is a unique noninvasive form of brain stimulation that modulates the internal nerve oscillation by delivering a low-intensity sinusoidal alternating current to the scalp (Elyamany et al., 2021), forcing the membrane potential oscillation away from its resting potential to slightly increased depolarization or hyperpolarization (Wu et al., 2021). In the depolarization state, neurons are more likely to respond to other neurons; this reaction is called stochastic resonance (Fertonani and Miniussi, 2017). The final effect of this reaction is that the firing time of neurons is locked to the increased stimulation frequency (Del Felice et al., 2019). Therefore, tACS plays a regulatory rather than dominant role and has great potential in the analysis of the causal relationship between neural activity and behavior (Herrmann et al., 2013).

In terms of motor performance and motor learning, there are further limitations and gaps in the existing research related to the effects of tACS. For motor performance, Rumpf et al. found no change in motor performance after applying 10 or 20 Hz tACS with an intensity of 1 mA to stimulate the left primary motor cortex (M1) during the motion sequence task (Rumpf et al., 2019). In contrast, Miyaguchi et al. used different amplitudes for tACS stimulation but found significant differences in motor performance between groups and significantly reduced task errors (Miyaguchi et al., 2019). The results were in accordance with Del Felice et al.‘s study, even in patients with Parkinson’s disease (Del Felice et al., 2019).

For motor learning, Krause et al. found that 10 Hz tACS stimulation can significantly reduce the reaction time and enhance motor learning ability after applying 1 mA, 10 Hz, or 20 Hz tACS stimulation to the left M1 (Krause et al., 2016). Antal et al. applied 1, 10, 15, 30, and 45 Hz tACS stimulation to the left M1. Transcranial magnetic stimulation (TMS) was used to detect the changes in spinal cord excitability before and after the reaction time task (RTT). Excitability significantly increased with only 10 Hz tACS stimulation, which indicated that 10 Hz tACS stimulation could improve motor learning (Antal et al., 2008). These results may be associated with stimulus parameters.

In view of the above, the efficacy of tACS on motor performance and motor learning skill needs to be explored. The present study systematically evaluated the effectiveness of tACS on motor performance and motor learning to determine the optimal treatment parameters of tACS for healthy adults, with the expectation of enriching the application of tACS in sports science.

## 2 Methods

Consistent with the preferred reporting items for systematic reviews and meta-analyses (PRISMA) statement (Moher et al., 2009), we conducted a systematic review and meta-analysis, which were registered with PROSPERO (registration number: CRD42022342884).

### 2.1 Inclusion criteria

The inclusion criteria strictly followed participants, interventions, controls, outcomes, and study design (PICOS) principles: 1) Participants: healthy adults; 2) Intervention: tACS; 3) Control: sham stimulation of tACS; 4) Outcomes: related outcome indicators of motor function [outcomes related to motor performance: task error, number of parts, accuracy, error rate (ER), time on target, seated chest pass throw (SCPT), seated backward overhead medicine ball throw (SBOMBT), squat jump (SJ), counter-movement jump (CMJ), counter-movement jump arm-swing (CMJ-AS); outcome related to motor learning: reaction time (RT)]; 5) Study design: random experiment, parallel, or cross-experimental design. Review articles, case studies, and animal studies were all excluded.

### 2.2 Source of information

As of 16 October 2022, the Cochrane Library, PubMed, EMBASE, and Web of Science were searched without using filters.

### 2.3 Search strategy

We searched using the following keywords: “transcranial alternating current stimulation” OR “tACS” OR “HD-tACS” and “motor behavior” OR “motor performance” OR “resistance” OR “strength” OR “weight” OR “power” OR “training” OR “training” OR “motor learning” OR “sequence learning” OR “motor skill” OR “motor acquisition”.

### 2.4 Study selection

Kun Hu and Ruihan Wan oversaw the initial screening *via* titles and abstracts from the electronic databases, independently and jointly excluding unrelated studies by reading titles and abstracts. For articles with unclear relevance, their full texts were retrieved to further evaluate their relevance. Then, all included articles were divided into relevant, possible, or irrelevant. The two authors determined the extent to which related research was subject to the PICOS principle. Finally, they carefully judged possible related studies and excluded unrelated studies. The places with different opinions were finally decided by the third author (Feng Guo).

### 2.5 Data extraction

The form on extracted information was jointly designed by two authors (Kun Hu and Ruihan Wan). The main extracted contents were as follows: characteristics of the participants (sample size, age, gender and group), tACS intervention program (electrode size, electrode position, intensity, and stimulus mode), exercise program (isometric force task, physical task, and so on), and main results (task error, accuracy, and so on). All the contents were extracted by the two authors. If the relevance was unclear, then the two authors would have a discussion to further reduce the risk of data extraction bias.

### 2.6 Quality assessment

Risk of bias was assessed in accordance with the criteria set out in the Cochrane Guidelines (Higgins and Green, 2011) (a) random sequence generation; (b) allocation concealment; (c) blinding of participants and personnel; (d) blinding of the outcome assessments; (e) incomplete outcome data; (f) selective reporting; and (g) other biases. The two evaluators (Kun Hu and Ruihan Wan) evaluated the eligible studies independently, and divided these into low, high, and unclear risk prejudices on the basis of the criteria (Higgins and Green, 2011). Any disagreement was discussed by the two evaluators and finally decided by the third author (Feng Guo).

### 2.7 Quantitative analysis

Review Manager software (RevMan 5.3; Cochrance Collaboration) was used for quantitative analysis. The main contents were as follows: subject characteristics, eligibility criteria, intervention programs, and main outcomes. Then, the heterogeneity of the study was evaluated to determine whether it was suitable for comprehensive analysis. To make the content of studies with large differences more comparable, the random-effects model was applied. The standardized mean difference (SMD) and 95% confidence interval (95% CI) were used to avoid the different measurement units of the data in the extracted research. Heterogeneity was evaluated by using chi-squared statistics (Chi^2^) and the heterogeneity index (I^2^). Significant heterogeneity exists when I^2^ is greater than 50% (Higgins and Green, 2011). The sources of heterogeneity were identified through subgroup analysis. Finally, sensitivity analysis was applied to exclude low-quality studies. In addition to evaluating the heterogeneity between studies, all values were analyzed by two-tailed analysis with a significance level of 5% (The *p*-value of the final result was calculated by RevMan 5.3. If p˂0.05, it indicated that tDCS had a significant effect on motor performance and motor learning.).

## 3 Results

**FIGURE 1:**
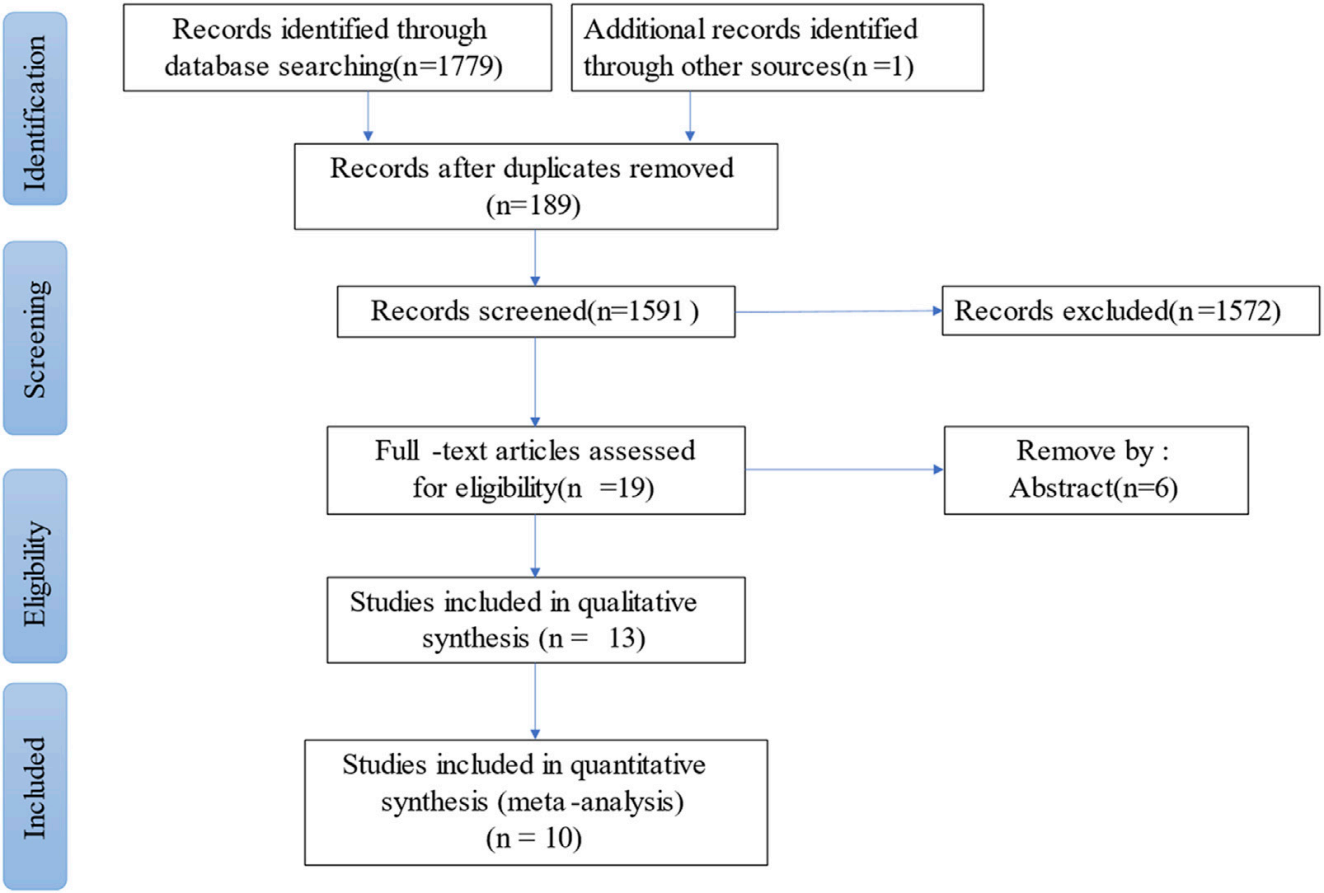
Study flow diagram.

A total of 1780 articles were initially screened from the electronic databases. After removing duplicate articles (n = 189) and relevant articles (n = 1,591), 19 articles were retained based on titles and abstracts. Articles that did not meet the inclusion criteria were excluded by browsing the full text (n = 6), including for qualitative analysis (n = 13). Ten eligible articles were analyzed quantitatively, as shown in Figure 1.

### 3.1 Research characteristics

Table 1 provides a detailed description of the participants’ characteristics of the effects of tACS on motor performance and motor learning. A total of 270 participants were involved in this analysis (170 males [63%]; 100 females [37%]), and the average age was between 20.7 ± 0.75 (Miyaguchi et al., 2019) and 66.8 ± 5.7 (Rumpf et al., 2019) years old. The control group of all studies was the sham group, and the experimental group of six studies (60%) was divided into two groups—alpha (*a*) (10 Hz), beta (*ß*) (20 Hz) group (Krause et al., 2016; Rumpf et al., 2019), or β (20 Hz), gamma (*?*) (>30 Hz) group (Miyaguchi et al., 2018; Miyaguchi et al., 2020; Giustiniani et al., 2021; Ma et al., 2021)—according to stimulus frequencies; three studies (30%) had one experimental group (γ group) (Miyaguchi et al., 2019; Miyaguchi et al., 2019; Miyaguchi et al., 2022); one (10%) study also had one experimental group (β group) (Yamaguchi et al., 2020).

**TABLE 1:** Demographic information of the participants.

References	N	Group	Female/Male	Age^b^
Miyaguchi et al. (2019) ^a^	20	70Hz/sham	6/14	21.3 ± 1
Miyaguchi et al. (2020)	32	20Hz/80Hz/sham	0/32	21.3 ± 1.5
Rumpf et al. (2019)	33	10Hz/20Hz/sham	10 Hz:9/720 Hz:14/3	10 Hz:68.5 ± 5.220 Hz:66.8 ± 5.7
Giustiniani et al. (2021)	17	50Hz/sham	50 Hz:7/4Sham:3/3	27.29 ± 10.65
Miyaguchi et al. (2018)	20	20Hz/70Hz/sham	0/20	21.5 ± 1.7
Ma et al. (2021)	50	20Hz/70Hz/sham	20 Hz:15/1470 Hz:6/15	20 Hz:22.1 ± 2.0270 Hz:22.43 ± 2.25
Krause et al. (2016)	36	10Hz/20Hz/sham	10 Hz:5/720 Hz:4/8Sham:5/7	10 Hz:26.17 ± 1.1820 Hz:26.42 ± 1.18Sham:25.33 ± 0.94
Miyaguchi et al. (2019) ^a^	20	70Hz/sham	6/14	20.7 ± 0.75
Miyaguchi et al. (2022)	22	70Hz/sham	10/12	21.3 ± 1.5
Yamaguchi et al. (2020)	20	Beta-band/sham	Beta-band:5/5 Sham:5/5	Beta-band: 24.1 ± 1.7Sham:5/524.7 ± 2.5

^a^ is used to distinguish the author who published two articles in the same year.

^b^ Data are represented as the means±SDs.

Table 2 describes the research characteristics of the impact of tACS on motor performance and motor learning in detail. All the included studies were randomized, among which 6 articles (60%) were crossover studies (Miyaguchi et al., 2018; Miyaguchi et al., 2019; Miyaguchi et al., 2019; Miyaguchi et al., 2020; Ma et al., 2021; Miyaguchi et al., 2022) and four articles (40%) were parallel studies (Krause et al., 2016; Rumpf et al., 2019; Yamaguchi et al., 2020; Giustiniani et al., 2021). Eight studies (80%) were related to motor performance (Miyaguchi et al., 2018; Miyaguchi et al., 2019; Miyaguchi et al., 2019; Rumpf et al., 2019; Yamaguchi et al., 2020; Giustiniani et al., 2021; Miyaguchi et al., 2020; Miyaguchi et al., 2022), and 2 (20%) studies were related to motor learning (Krause et al., 2016; Ma et al., 2021). With regard to the exercise program included in the study, the exercise tasks of all studies in motor performance were inconsistent, including visuomotor control task (VCT), visuomotor tacking (VMT), Purdue Pegboard Test (PPT), motor sequence learning task (MSLT), physical test (PT), and isometric force task (IFT); in motor learning, all the research tasks were RTTs. The stimuli in the seven studies were located at the left M1 (Krause et al., 2016; Miyaguchi et al., 2018; Miyaguchi et al., 2019; Miyaguchi et al., 2019; Rumpf et al., 2019; Yamaguchi et al., 2020; Ma et al., 2021); one study simultaneously stimulated the left and right M1 (Giustiniani et al., 2021); the stimulation position of two studies was the supplementary motor area (SMA) (Miyaguchi et al., 2020; Miyaguchi et al., 2022). The stimulation intensity was 1 mA (Krause et al., 2016; Miyaguchi et al., 2018; Miyaguchi et al., 2019; Miyaguchi et al., 2019; Rumpf et al., 2019; Miyaguchi et al., 2020; Miyaguchi et al., 2022), 1.5 mA (Giustiniani et al., 2021) and 2 mA (Yamaguchi et al., 2020), and the area of the stimulation electrode was 25 cm^2^ (Miyaguchi et al., 2018; Miyaguchi et al., 2019; Miyaguchi et al., 2019; Miyaguchi et al., 2020; Giustiniani et al., 2021; Miyaguchi et al., 2022) and 35 cm^2^ (Krause et al., 2016; Rumpf et al., 2019; Yamaguchi et al., 2020). We could not determine the intensity and area of stimulation in Ma’s study (Ma et al., 2021). The stimuli in the six studies (60%) were online stimuli (Miyaguchi et al., 2018; Miyaguchi et al., 2019; Miyaguchi et al., 2019; Miyaguchi et al., 2020; Ma et al., 2021; Miyaguchi et al., 2022); the stimuli in the four studies (40%) were offline stimuli (Krause et al., 2016; Rumpf et al., 2019; Yamaguchi et al., 2020; Giustiniani et al., 2021).

**TABLE 2:** Characteristics of the included studies.

References	Design	Task	Outcomes	Target	Intesity (mA)	Electrode size (cm^2^)	Stimulus mode
Miyaguchi et al. (2019) ^a^	Crossover	VCT	Task error	Left M1	1	25	online
Miyaguchi et al. (2020)	crossover	PPT	Number of parts	SMA	1	25	online
Rumpf et al. (2019)	RCT	MSLT	Accuracy	Left M1	1	35	offline
Giustiniani et al. (2021)	RCT	PT	SCPT/SBOMBT/SJ/CMJ/CMJ-AS	Left and right M1	1.5	25	offline
Miyaguchi et al. (2018)	crossover	IFT	ER	Left M1	1	25	online
Ma et al. (2021)	crossover	RTT	Accuracy	Left M1	N/D	N/D	online
Krause et al. (2016)	RCT	SRTT	RT	Left M1	1	35	offline
Miyaguchi et al. (2019) ^a^	crossover	VCT	Task error	Left M1	1	25	online
Miyaguchi et al. (2022)	crossover	PPT	Number of parts	SMA	1	25	online
Yamaguchi et al. (2020)	RCT	VMT	Time to target	Left M1	2	35	offline

^a^ is used to distinguish when the author published two articles in the same year.

*RCT* = randomized controlled trial, *VCT* = Visuomotor control task, *PPT* = Purdue Pegboard Test, *MSLT* = Motor sequence learning task, *PT* = Physical test, *IFT* = Isometric force task, *RTT* = Reaction time task, *SRTT* = Serial reaction time task, *VMT* = Visuomotor tracking, *SCPT* = Seated chest pass throw, *SBOMBT* = Seated backward overhead medicine ball throw, *SJ* = Squat jump, *CMJ* = Countermovement jump, *CMJ-AS* = Countermovement jump with arm swing, *ER* = Error rate, *RT* = Reaction time, *M1* = Primary motor cortex, *SMA* = Supplementary motor area, *N/D* = Not describe.

### 3.2 Main results and quantitative synthesis

#### 3.2.1 Effect of tACS on motor performance

**FIGURE 2:**
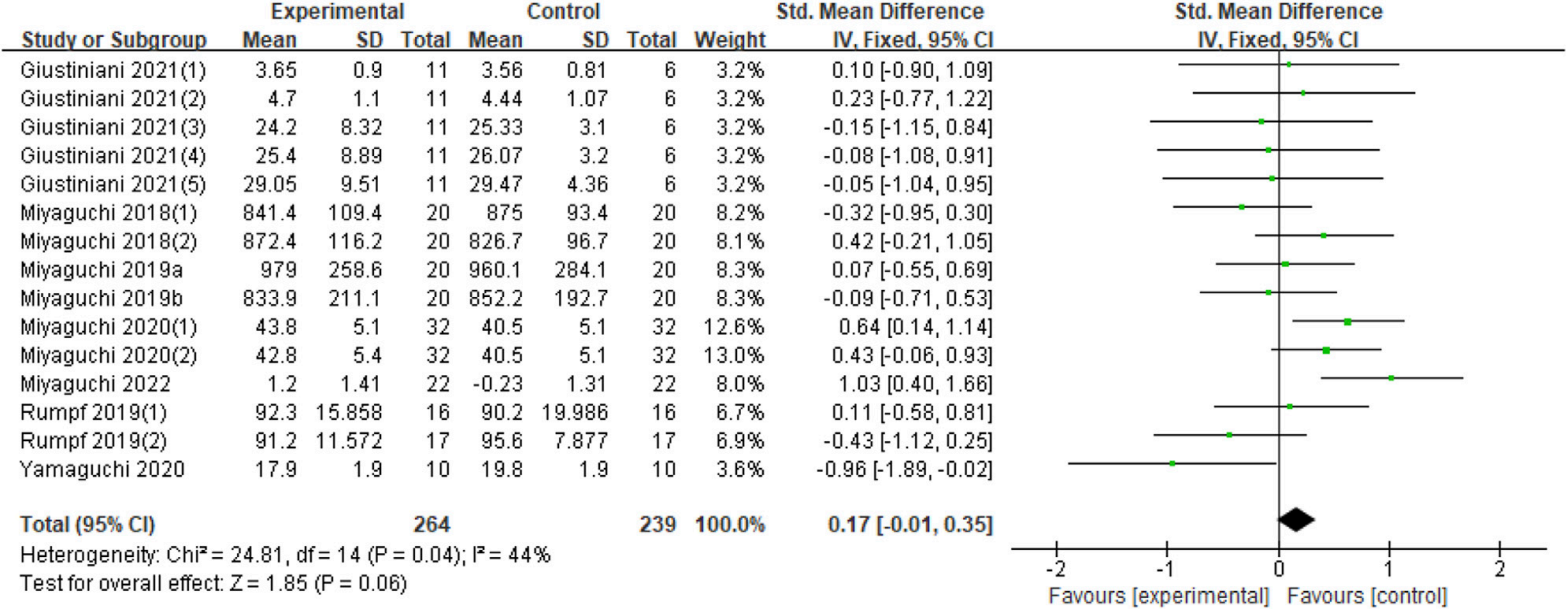
Forest plot for the effect of tACS on improving motor performance. Notes: Giustiniani (1) (2) (3) (4) (5)-The five outcome measures in Giustiniani’s study were SCPT, SBOMBT, SJ, CMJ, CMJ-AS; Miyaguchi 2018 (1) (2)- The 20 Hz and 70 Hz stimuli in this study, respectively; Miyaguchi 2020 (1) (1)- The 20 Hz and 80 Hz stimuli in this study, respectively; Rumpf (1) (2)- The 10 Hz and 20 Hz stimuli in this study, respectively; Miyaguchi et al. published two studies in 2019 distinguished by a and **(B)**

In terms of the impact of tACS on motor performance, eight studies were included in this analysis (Miyaguchi et al., 2018; Miyaguchi et al., 2019; Miyaguchi et al., 2019; Rumpf et al., 2019; Miyaguchi et al., 2020; Yamaguchi et al., 2020; Giustiniani et al., 2021; Miyaguchi et al., 2022). The outcomes of each study were inconsistent. Giustiniani et al. included five outcomes (Giustiniani et al., 2021) from seated chest pass throw (SCPT), seated backward overhead medicine ball throw (SBOMBT), squat jump (SJ), counter-movement jump (CMJ), and counter-movement jump arm-swing (CMJ-AS). The outcome indicators of the other seven studies are task error, number of parts, accuracy, time of target and error rate (Miyaguchi et al., 2018; Miyaguchi et al., 2019; Miyaguchi et al., 2019; Rumpf et al., 2019; Miyaguchi et al., 2020; Yamaguchi et al., 2020; Miyaguchi et al., 2022). The experimental groups in most studies included two groups, namely, the α, β, or β, γ groups (Miyaguchi et al., 2019; Rumpf et al., 2019; Miyaguchi et al., 2020; Giustiniani et al., 2021); three experimental groups were γ groups (Miyaguchi et al., 2018; Miyaguchi et al., 2019; Miyaguchi et al., 2022); and only one experimental group was used by Yamaguchi, which is the β group (Yamaguchi et al., 2020). Therefore, a total of 15 datasets were included in this synthesis. Compared with the sham group, no significant effect was found on the improvement of exercise performance for application of tACS (Z = 1.85, *p* = 0.06). Nevertheless, moderate heterogeneity (Chi^2^ = 24.81, *p* = 0.04, I^2^ = 44%) was observed. Thus, quantitative synthesis could be included (Figure 2).

#### 3.2.2 Influence of tACS with different frequencies on motor performance

The tACS studies included in this meta-analysis were mainly divided into three different stimulus frequencies (alpha, beta, and gamma bands). Among the 15 datasets, only one dataset was the alpha (10 Hz) band (one dataset could not be included in the synthetic analysis), and the other datasets were the beta (20 Hz) and gamma (>30 Hz) bands. Therefore, we tried to incorporate tACS research at beta and gamma frequencies into quantitative synthesis, with a total of 14 datasets.

##### 3.2.2.1 β-tACS (20 Hz)

**FIGURE 3:**
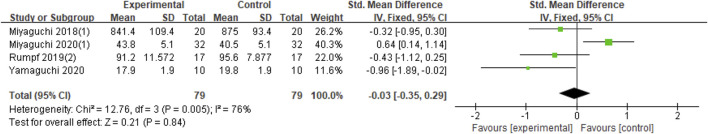
Forest plot for the effect of β-tACS on improving motor performance.

Four datasets (28.6%) were included in this study. Compared with the sham group, no significant effect was observed on the improvement of motor performance for β-tACS (Z = 0.21, *p* = 0.84) with more significant heterogeneity (Chi^2^ = 12.76, *p* = 0.005, I^2^ = 76%) (Figure 3).

##### 3.2.2.2 γ-tACS(>30 Hz)

**FIGURE 4:**
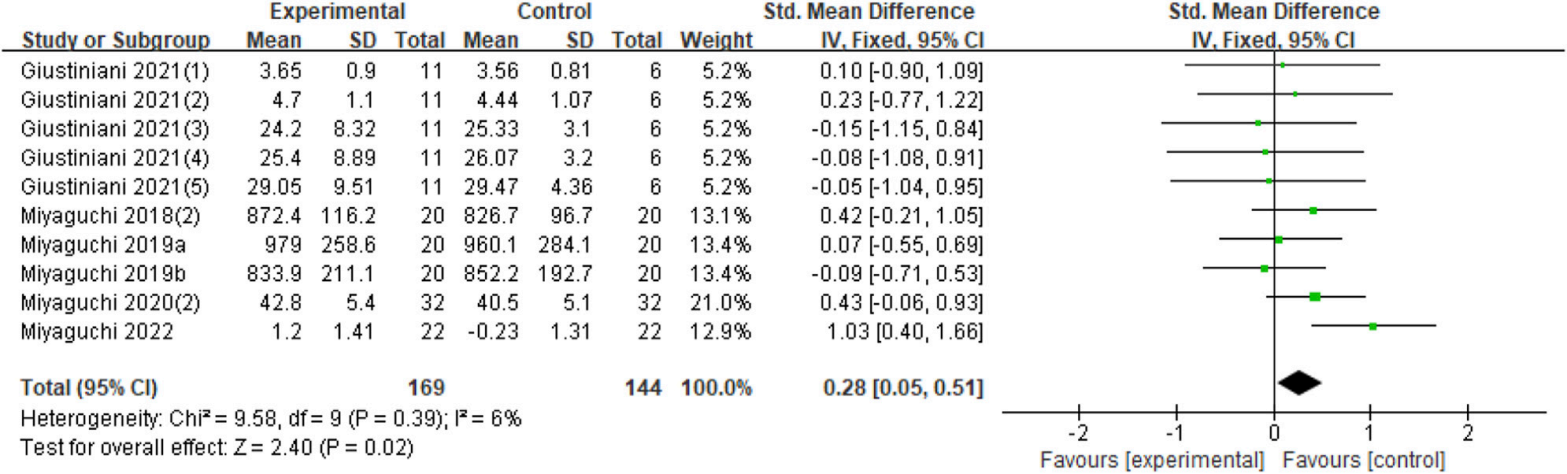
Forest plot for the effect of γ-tACS on improving motor performance.

Ten datasets (71.4%) were included in this study. Compared with the sham group, γ-tACS had significant effect on improving motor performance (Z = 2.40, *p* = 0.02), and had good heterogeneity (Chi^2^ = 9.58, *p* = 0.39, I^2^ = 6%) (Figure 4).

#### 3.2.3 Effects of different tACS stimulation modes on motor performance

The studies included in this meta-analysis were mainly divided into two types of stimuli (online and offline). Fifteen datasets were included in the quantitative synthesis.

##### 3.2.3.1 Online

**FIGURE 5:**
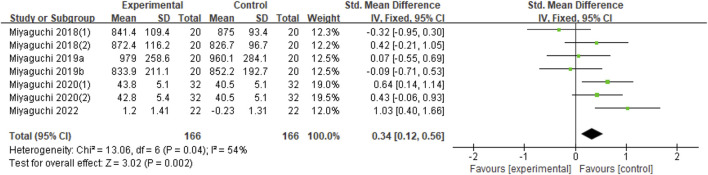
Forest plot for the effect of online stimulation on improving motor performance.

Seven datasets were included in the synthetic analysis. Compared with the sham group, online stimulation significantly improved motor performance (Z = 3.02, *p* = 0.002) but with moderate heterogeneity (Chi^2^ = 13.06, *p* = 0.04, I^2^ = 54%) (Figure 5).

##### 3.2.3.2 Offline

**FIGURE 6:**
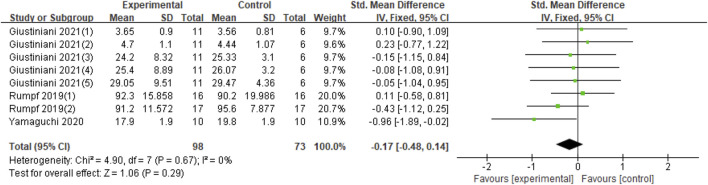
Forest plot for the effect of offline stimulation on improving motor performance.

For meta-analysis, eight datasets were included in the quantitative synthesis. Compared with the sham group, offline stimulation did not improve motor performance (Z = 1.06, *p* = 0.29) and had no significant heterogeneity (Chi^2^ = 4.90, *p* = 0.67, I^2^ = 0%) (Figure 6).

#### 3.2.4 Influence of tACS on motor learning

**FIGURE 7:**
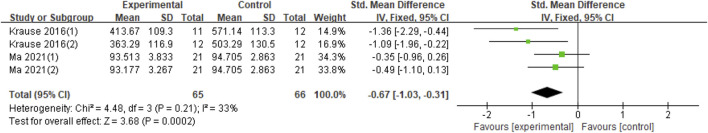
Forest plot for the effect of tACS on improving motor learning. Notes: Krause (1) (2)- The 10 Hz and 20 Hz stimuli in this study, respectively; Ma (1) (2)- The 20 Hz and 70 Hz stimuli in this study, respectively.

With regard to the effect of tACS on motor learning, this analysis included two studies. The experimental groups in both studies had two groups; thus, the experimental group in Krause’s study was α and β-tACS stimulation (Krause et al., 2016); in the study of Ma (Ma et al., 2021), β and γ stimuli were used in the experimental group. Four datasets are therefore included in this synthesis. Compared with the sham group, tACS significantly improved motor learning ability (Z = 3.68, *p* = 0.0002) but had moderate heterogeneity (Chi^2^ = 4.48, *p* = 0.21, I^2^ = 33%) (Figure 7).

##### 3.2.4.1 Risk of bias in included studies

**FIGURE 8:**
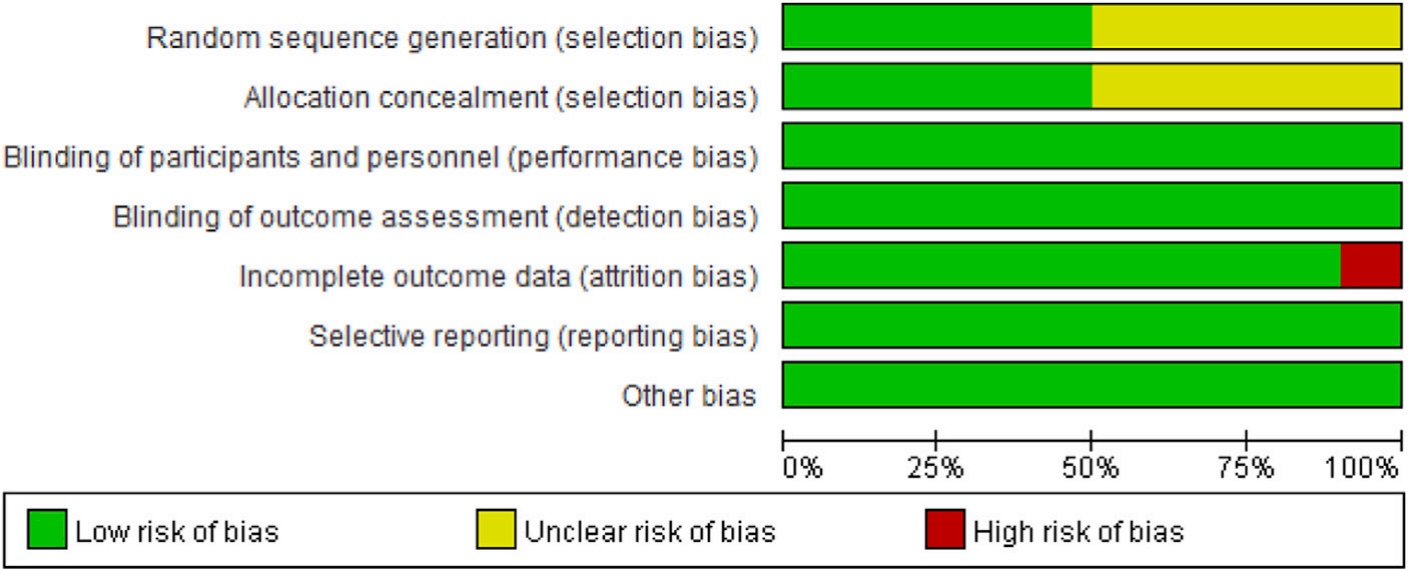
Risk of bias in included studies.

As shown in Figure 8, reporting bias (selective report results), detection bias (blinding of the outcome assessments), performance bias (blinding of participants and personnel), and other biases were all low-risk biases. For random sequence generation and allocation concealment, Miyaguchi (2018), Miyaguchi (2019) ^a^, Miyaguchi (2019) ^b^, Miyaguchi (2020) and Miyaguchi (2022) did not report these biases (Miyaguchi et al. published two studies in 2019, which we distinguished by a and b) (Miyaguchi et al., 2018; Miyaguchi et al., 2019; Miyaguchi et al., 2019; Miyaguchi et al., 2020; Miyaguchi et al., 2022); thus, they were unclear risk biases. In addition, in the attrition bias (incomplete outcome data), one subject was dropped from the experiment in Rumpf’s study, resulting in high-risk bias being reported (Rumpf et al., 2019).

## 4 Discussion

To our knowledge, the present study was the first to systematically evaluate the efficacy of tACS on motor performance and motor learning in healthy individuals. Significant conclusions can be shown that tACS can effectively improve motor performance (beta band and online mode) and motor learning in healthy individuals compared with the sham group.

Although the mechanism of tACS action remains unclear, previous studies have shown that entrainment of stimulation frequencies by brain oscillations and coupling or decoupling of distant oscillatory connections between distant brain regions may be involved (Weinrich et al., 2017; Schwab et al., 2019; Elyamany et al., 2021). Therefore, the effectiveness of tACS on motor performance in the current study was divided into two main categories, including online effects (during the stimulation period) and offline effects (outlast the stimulation period); Considering the difference in stimulation frequency, the current study classified it into three categories—α (10 Hz), β (20 Hz), and γ-tACS (>30 Hz) —to analyze on the premise of roughly the same stimulation position and intensity.

Although the synthesis results showed low heterogeneity (Figure 2), the uncertainty of the synthesis of the study can be diminished. In the 15 datasets of eight studies, only one dataset was α-tACS; β-tACS and γ-tACSs are mainstream, consistent with the current research status. The current belief is that β-tACS and γ-tACS stimuli affect motor performance and motor learning (Pogosyan et al., 2009; Joundi et al., 2012; Pollok et al., 2015; Moisa et al., 2016; Santarnecchi et al., 2017). Therefore, we mainly discussed β-tACS and γ-tACSs.

In terms of motor performance, as shown in Figure 3, β-tACS did not improve motor performance in healthy individuals and presented a significant heterogeneity, which may be linked to the differences in exercise program, tACS stimulation location, and stimulation mode. Notably, Miyaguchi et al. (2020) found that β-tACS was positively correlated with exercise performance and γ-tACS was negatively correlated with motor performance. These results were in line with Muthukumaraswamy’s study (Muthukumaraswamy et al., 2011). β-band activity was also deemed anti-dynamic activity, and γ-band activity was essentially pro-dynamic activity (Miyaguchi et al., 2018). These findings indicate that different stimulus frequencies played a major role in motor performance (Miyaguchi et al., 2020). In contrast, γ-tACS resulted in improved motor performance, and the results were not significantly heterogeneous (Figure 4). Interestingly, ten datasets of six studies were included, and five datasets were from the same study. The other five datasets come from five studies by the same team: Miyaguchi (2018), Miyaguchi (2019) ^a^, Miyaguchi (2019) ^b^ Miyaguchi (2020) and Miyaguchi (2022) (Miyaguchi et al. published two studies in 2019, which we distinguished by a and b) (Miyaguchi et al., 2018; Miyaguchi et al., 2019; Miyaguchi et al., 2019; Miyaguchi et al., 2020; Miyaguchi et al., 2022). The effect of tACS stimulation at different frequencies (70 Hz or 80 Hz) on motor performance was significantly different, which was not reflected by Giustiniani et al., who applied a frequency of 50 Hz (Giustiniani et al., 2021). This result may have occurred because 50 Hz tACS was thought to increase the speed of visual motion (Moisa et al., 2016) and time-dependent modulation of γ-aminobutyric acid (Nowak et al., 2017), indicating that tACS stimuli in the γ-band have great frequency specificity. Further attention should be given to the detailed frequency study of γ-tACS.

In relation to the stimulation of tACS on motor performance, online tACS can significantly improve the motor performance of healthy individuals (Figure 5). No significant effect was observed on offline tACS for motor performance, which indicated that the stimulation mode was the main influencing factor in the study of tACS on motor performance.

At present, it remains controversial whether tACS plays a role during or after stimulation (post-effect) (Fertonani et al., 2017; Samaei et al., 2017; Galli et al., 2019). TMS-induced motor-evoked potential (MEP) measurement of tACS online and offline effects has been widely recognized (Pozdniakov et al., 2021). Growing evidence has confirmed the frequency-specific online effect of tACS through TMS-induced MEP (Feurra et al., 2011; Feurra et al., 2013; Shpektor et al., 2017; Feurra et al., 2019). For example, Feurra et al. explored the effect of online tACS stimulation at different frequencies on the spinal cord excitability of M1 *via* TMS and found that 20 Hz tACS stimulation increased the excitability of the cortical spinal cord, while 5, 10, and 40 Hz had no effect on MEP (Feurra et al., 2011), which was also verified in Feurra et al.‘s two studies (Feurra et al., 2013; Feurra et al., 2019). In addition, no consistent conclusion was derived on offline tACS. Antal et al. explored the changes in spinal cord excitability of the motor cortex through TMS-induced MEP, and no significant change was found in spinal cord excitability when stimulated by tACS at 1, 10, 15, 30, and 45 Hz after intergroup analysis (Antal et al., 2008). Heise et al. evaluated spinal cord excitability before, during, and after stimulation at 20 Hz tACS and found that spinal cord excitability increased significantly during immediately after stimulation (Heise et al., 2016). These results show that tACS stimulation seems to have a certain timeliness. Hence, although online tACS achieves a significant improvement in motor performance, future research still needs to focus on the timeliness of tACS, which will have far-reaching significance for the standardization of clinical application.

In terms of motor learning, as shown in Figure 7, a significant improvement was found in healthy individuals. The analysis included only four datasets of two studies; thus, the lack of sample size was a key problem. In addition, inconsistent with a previous study, only one of the four datasets was γ-tACS, which made determining the role of tACS in motor learning ability impossible. Sugata et al. and Giustiniani showed the potential of the high-frequency band γ-tACS on sequential learning tasks (Sugata et al., 2018; Giustiniani et al., 2019). Transcranial electrical stimulation (TES) has a good regulating effect on neuroplasticity (Galea et al., 2009; Zuchowski et al., 2014; Wessel et al., 2016), and tACS can exert a more selective effect on target neurons, so that the “characteristic frequency” of neurons tends to the stimulation frequency (Antal et al., 2016; Naro et al., 2017). In addition, previous studies have found that oscillatory activity in gamma and beta bands plays an important role in motor learning (Pollok et al., 2014; Pollok et al., 2015; Naro et al., 2016; Naro et al., 2017; Nowak et al., 2017; Wessel et al., 2020). These oscillatory activities are thought to be induced by the activation of excitatory glutamatergic cells and inhibitory GABAergic interneurons in M1 (Buzsáki et al., 2012; Guerra et al., 2016). Among them, Naro and Wessel found that a 50 Hz stimulation frequency can promote motor learning ability (Naro et al., 2016; Naro et al., 2017; Wessel et al., 2020). In accordance with a previous study, α and β-tACSs could significantly improve motor learning ability (Pollok et al., 2014; Sugata et al., 2014; Pollok et al., 2015; Krause et al., 2016). Among them, alpha oscillations affect vision and sensorimotor activity (Sugata et al., 2014); beta oscillations affect motor performance and motor learning (Houweling et al., 2008; Sugata et al., 2014). In brief, γ-tACS should receive more attention for motor learning, especially the impact of frequency specificity.

## 5 Limitations

This systematic review and meta-analysis has several limitations. First, some methodological variables of this study were not standardized and unified, particularly the stimulation frequency and stimulation mode (online and offline), which may directly affect the accuracy and reliability of the results of this study. There were no significant findings in this area; thus, a more precise experimental design should be conducted to systematically standardize this problem in the future. Second, few quantitative analyses were included, which increased the incidence of false negative or false positive synthesis results. Therefore, the authors are cautious about the results of these tACS analyses on motor learning. More research evidence is needed to support our results in the future. Third, this study did not report the safety issues of tACS (skin sensation, phosphenes, other sensation, *etc.*). Thus, caution should be taken in the application of tACS in healthy people and even special populations. In a previous meta-analysis of tACS on cortical spinal cord excitability, the authors reported that tACS had certain effects on skin sensation and perceiving phosphenes in healthy adults (Wischnewski et al., 2019). Therefore, safe application of tACS in healthy people and even special populations is needed clinically. The physiological mechanism by which tDCS improves motor performance and motor learning is still unknown. At present, the most widely used technology to observe spinal cord excitability during stimulation is TMS-induced MEP, but this technology still has certain limitations. The authors suggest that future research should combine tACS technology with neuroimaging technology. For example, simultaneous use of electroencephalogram (EEG) with high temporal resolution can immediately explore brain changes during tACS stimulation and reveal the physiological mechanism of tACS. Combined with functional near-infrared spectroscopy (fNIRS), which has low movement requirements and small movement artifacts, it can meet more task designs and reveal the physiological mechanism of tACS application in certain groups (such as high-level athletes, dyskinesia patients, hyperkinesia patients, *etc.*).

## 6 Conclusion

This systematic review and meta-analysis found that online tACS and gamma band tACS can significantly improve motor performance. In addition, tACS has a certain effect on motor learning, but the authors remain cautious about this conclusion. In the future, more research evidence is needed to verify the efficacy of tACS on motor learning.

Abr7 Abbreviations: tACS-transcranial alternating current stimulation, M1-primary motor cortex, TMS-transcranial magnetic stimulation, RTT-reaction time task, SRTT-Serial reaction time task, SMD-standardized mean difference, SMA-supplementary motor area, SCPT-seated chest pass throw, SBOMBT-seated backward overhead medicine ball throw, SJ-squat jump, CMJ-counter-movement jump, CMJ-AS-counter-movement jump arm-swing, MEP-motor-evoked potential, TES-transcranial electrical stimulation, EEG-electroencephalogram, fNIRS-functional near-infrared spectroscopy, RCT-Randomized controlled trial, VCT-Visuomotor control task, PPT-Purdue Pegboard Test, MSLT-Motor sequence learning task, PT-Physical test, IFT-Isometric force task, VMT-Visuomotor tracking, ER-Error rate, RT-Reaction time.

## Data Availability

The original contributions presented in the study are included in the article/supplementary materials, further inquiries can be directed to the corresponding author.
